# The Role of the Microbiota in Graves’ Disease and Graves’ Orbitopathy

**DOI:** 10.3389/fcimb.2021.739707

**Published:** 2021-12-22

**Authors:** Jueyu Hou, Yunjing Tang, Yongjiang Chen, Danian Chen

**Affiliations:** ^1^ The Research Laboratory of Ophthalmology and Vision Sciences, State Key Laboratory of Biotherapy, West China Hospital, Sichuan University, Chengdu, China; ^2^ The Department of Ophthalmology, West China Hospital, Sichuan University, Chengdu, China; ^3^ The School of Optometry and Vision Science, University of Waterloo, Waterloo, ON, Canada

**Keywords:** gut microbiota, Graves’ disease, Graves’ orbitopathy (GO), TSHR (thyroid-stimulating hormone receptor), Th17 and Treg cells, *Lactobacillus*, *Prevotella*, *Veillonella*

## Abstract

Graves‘ disease (GD) is a clinical syndrome with an enlarged and overactive thyroid gland, an accelerated heart rate, Graves’ orbitopathy (GO), and pretibial myxedema (PTM). GO is the most common extrathyroidal complication of GD. GD/GO has a significant negative impact on the quality of life. GD is the most common systemic autoimmune disorder, mediated by autoantibodies to the thyroid-stimulating hormone receptor (TSHR). It is generally accepted that GD/GO results from complex interactions between genetic and environmental factors that lead to the loss of immune tolerance to thyroid antigens. However, the exact mechanism is still elusive. Systematic investigations into GD/GO animal models and clinical patients have provided important new insight into these disorders during the past 4 years. These studies suggested that gut microbiota may play an essential role in the pathogenesis of GD/GO. Antibiotic vancomycin can reduce disease severity, but fecal material transfer (FMT) from GD/GO patients exaggerates the disease in GD/GO mouse models. There are significant differences in microbiota composition between GD/GO patients and healthy controls. *Lactobacillus*, *Prevotella*, and *Veillonella* often increase in GD patients. The commonly used therapeutic agents for GD/GO can also affect the gut microbiota. Antigenic mimicry and the imbalance of T helper 17 cells (Th17)/regulatory T cells (Tregs) are the primary mechanisms proposed for dysbiosis in GD/GO. Interventions including antibiotics, probiotics, and diet modification that modulate the gut microbiota have been actively investigated in preclinical models and, to some extent, in clinical settings, such as probiotics (*Bifidobacterium longum*) and selenium supplements. Future studies will reveal molecular pathways linking gut and thyroid functions and how they impact orbital autoimmunity. Microbiota-targeting therapeutics will likely be an essential strategy in managing GD/GO in the coming years.

## Introduction

Graves’ disease (GD) is an autoimmune disorder characterized by the unique association of an enlarged and overactive thyroid gland, an accelerated heart rate, Graves’ orbitopathy (GO), and Graves’ dermopathy such as pretibial myxedema (PTM), in its typical presentation. GD is the most common cause of hyperthyroidism. The lifetime risk is about 3% for women and 0.5% for men (Davies et al., 2020). GD is mediated by autoantibodies to the thyroid-stimulating hormone receptor (TSHR). The TSHR is also expressed in orbital fibroblasts. GO is the most common extrathyroidal complication of GD (Covelli and Ludgate, 2017). About 25%–30% of GD patients have GO, but careful orbital imaging analysis can identify subtle orbital soft tissue abnormalities in 50%–70% of GD patients (Smith and Hegedüs, 2016; Perros et al., 2017; Taylor et al., 2020). The annual GO incidence is about 16 cases per 100,000 Europeans and 10 cases per 100,000 Japanese (Hiromatsu et al., 2014; Perros et al., 2017). GO is characterized by orbital tissue remodeling, retro-orbital inflammation, and glycosaminoglycan accumulation (Hansen et al., 1999). The major clinical features include periorbital edema, eyelid lag, proptosis, limited ocular movement, orbital disfigurement, and diplopia (Ludgate, 2020). Glucocorticoids are the primary treatment for GO at the active stage; smoking cessation, selenium supplements, and ocular lubricants are also helpful. Approximately 2% of GO patients will develop moderate to severe disease. These patients can have a visual loss due to corneal ulcers or GO-related optic neuropathy and eventually need decompression surgery (Davies et al., 2020). Thus, GO has a significant negative impact on the quality of life (Taylor et al., 2020).

GD is a multifactorial disease resulting from complex interactions between genetic and environmental factors that lead to the loss of immune tolerance to thyroid antigens. TSHR, T-cell-mediated immunity, and the mesenchymal stem cell properties of orbital fibroblasts have been implicated in the pathogenesis of GO (Taylor et al., 2020). However, the exact mechanism is still elusive. Some studies support the causative roles of microbiota in the pathogenesis of GD/GO (Kohling et al., 2017; Masetti and Ludgate, 2020).

Trillions of microorganisms exist on the mucosal and epidermal surfaces in the human body, such as skin, mouth, nose, sinuses, gut, respiratory tract, and ocular surface. These microbes are composed of bacteria, viruses, and fungi; usually do not harm; and are beneficial for the human body (Belkaid and Hand, 2014; Khan et al., 2019). The gastrointestinal tract is the primary interface in the human body to host microorganisms. Gut microbiota includes all microorganisms in the gastrointestinal mucosa and has about 10^14^ microbial cells (Szablewski, 2018). Gut microbiota can protect the host from pathogens, accelerate food digestion and mineral uptake (such as selenium, iron, and zinc), and modulate the immune system (Fröhlich and Wahl, 2019; Shivaji, 2019). Dysbiosis is an imbalance of the typical gut microbiota composition (Robles Alonso and Guarner, 2013). Dysbiosis can change the regulatory signaling of the immune system, resulting in pathological conditions of many organs (Gritz and Bhandari, 2015).

Indeed, increasing evidence has revealed that dysbiosis is closely connected to many diseases, including autoimmune diseases [e.g., rheumatoid arthritis (Gianchecchi and Fierabracci, 2019; Alghamdi and Redwan, 2021) and multiple sclerosis (Tsunoda, 2017; Zeng et al., 2019; Wang et al., 2021)], inflammatory diseases [e.g., ankylosing spondylitis (Ciccia et al., 2017; Wen et al., 2017; Berlinberg et al., 2021), infective endocarditis (Del Giudice et al., 2021), and inflammatory bowel disease (Kassam et al., 2018; Gianchecchi and Fierabracci, 2019; Pavel et al., 2021)], and ocular diseases [e.g., age-related macular degeneration (Rowan et al., 2017; Rinninella et al., 2018), diabetic retinopathy (Beli et al., 2018), dry eye (Cavuoto et al., 2019; Trujillo-Vargas et al., 2020), glaucoma (Chen et al., 2018; Doulberis et al., 2019), and uveitis (Fu et al., 2021)].

We will discuss the changes and potential mechanisms of the gut microbiota in the pathogenesis of GD/GO and comment on some possible therapeutic means to treat GD/GO by targeting the gut microbiome in this article.

## Changes of the Gut Microbiome in GD/GO

During the past 20 years, animal model studies have indicated a critical role of the gut microbiota in regulating innate and adaptive immune responses (Virili et al., 2021). Germ-free (GF) mouse models provide the most strong evidence to support that notion, including models of spontaneous ankylosing enteropathy (Rehakova et al., 2000), autoimmune arthritis (Wu et al., 2010), autoimmune encephalomyelitis (Lee et al., 2011), and autoimmune uveitis (Heissigerova et al., 2016; Fu et al., 2021). In these GF animal models, the disease incidence and severity are reduced under the GF environment, indicating the microbiota is crucial for the initiation and progression of these diseases (Vieira et al., 2014; Carding et al., 2015). This conclusion is further confirmed in clinical observations of many patients with ankylosing spondylitis (Ciccia et al., 2017; Wen et al., 2017), rheumatoid arthritis (Gianchecchi and Fierabracci, 2019), uveitis (Huang et al., 2018; Fu et al., 2021), and multiple sclerosis (Tsunoda, 2017; Zeng et al., 2019). GD is an autoimmune thyroid disease (AITD); the role of the gut microbiota in the pathogenesis of GD/GO was only discovered recently, based on both mouse models and clinical investigations (**Table 1**). These results are supported by findings that therapeutic agents of GD/GO (such as antithyroid drugs, glucocorticoids, immunosuppressants, and biologics) can also change the microbiota composition (**Table 2**).

**Table 1:** Major references connecting the gut microbiome with GD/GO.

Year	Study Type	Subjects	Major Findings	References
2018	Experimental	BALB/c female, two locations	Disease-associated taxonomies explain the GD/GO variations observed	Masetti et al. (2018)
2018	Experimental	BALB/c and C57BL/6J females	Big differences of BALB/c and C57BL/6J gut microbiota composition	Moshkelgosha et al. (2018)
2020	Experimental	BALB/c female, FMT	FMT from GD donor increased the severity of GD	Su et al. (2020)
2021	Experimental	BALB/c female, microbiota modification	Vancomycin reduced, but FMT from GO donor increased the severity of GD/GO	Moshkelgosha et al. (2021)
2018	Clinical-GD	27 GD/12 HC	Diversity reduced, F/B ratio increased	Ishaq et al. (2018)
2019	Clinical-GD	15 GD/15 HC	Diversity reduced, F/B ratio increased	Yang M. et al. (2019)
2020	Clinical-GD	9 GD/11 HC	Diversity reduced	Cornejo-Pareja et al. (2020)
2020	Clinical-GD	58 GD/63 HC	Diversity reduced	Su et al. (2020)
2020	Clinical-GD	39 GD/17 HC	Diversity reduced	Yan et al. (2020)
2021	Clinical-GD	15 GD/14 HC	Diversity reduced	Chen et al. (2021)
2021	Clinical-GD	55 GD/48 HC	Diversity unchanged, F/B ratio decreased	Chang et al. (2021)
2021	Clinical-GD	45 GD/59 HC	Diversity reduced, F/B ratio decreased	Jiang et al. (2021)
2019	Clinical-GO	33 GO/32 HC	Diversity reduced, F/B ratio decreased	Shi et al. (2019b)
2019	Clinical-GO	31 GO	Links between the gut microbiota and GO-related traits are identified	Shi et al. (2019a)
2021	Clinical-GD/GO	30 GD/33 GO/32 HC	Random forest algorithm can identify the three groups with 70–80% accuracy	Shi et al. (2021)

GD, Graves’ disease; GO, Graves’ orbitopathy; F/B ratio, Firmicutes/Bacteroidetes ratio; HC, healthy controls.

**Table 2:** Major references connecting the gut microbiome with therapeutic agents for GD/GO.

Year	Study Type	Therapeutic Agent	Subjects	References
2020	Experimental	PTU	Adult male SD rat	Shin et al. (2020)
2020	Experimental	PTU/MMI	Adult female SD rat	Sun et al. (2020)
2020	Clinical	PTU/MMI	GD patient	Sun et al. (2020)
2021	Clinical	MMI	GD patient	Chen et al. (2021)
2021	Clinical	MMI	GD patient	Huo et al. (2021)
2011	Experimental	Stress (increased steroid)	Mouse	Bailey et al. (2011)
2018	Experimental	GCs	Bird	Noguera et al. (2018)
2019	Clinical	GCs	Patient with GC-induced obesity	Qiu et al. (2019)
2020	Experimental	GCs (short term)	Mouse	Zhao et al. (2020)
2020	Experimental	GCs (long term)	Mouse	Schepper et al. (2020)
2021	Clinical	AZA	Crohn’s disease patient	Effenberger et al. (2021)
2018	Experimental	MMF	Mouse	Flannigan et al. (2018)
2021	Experimental	MMF	Spontaneously hypertensive rat (SHR)	Robles-Vera et al. (2021)
2019	Clinical	Anti-TNF-α antibody	Crohn’s disease patient	Yilmaz et al. (2019)
2021	Clinical	Anti-TNF-α antibody	Enteropathic arthritis patient	Ditto et al. (2021)
2021	Clinical	Anti-TNF-α antibody	Crohn’s disease patient	Effenberger et al. (2021)

AZA, azathioprine; GD, Graves’ disease; GCs, glucocorticoids; GO, Graves’ orbitopathy; MMI, methimazole; MMF, mycophenolate mofetil; PTU, propylthiouracil; TNF-α, antitumor necrosis factor-α.

### Animal Models of GD/GO

Previously, a GD/GO animal model was established by transferring human TSHR-primed T cells into female BALB/c mice (Many et al., 1999). It was initially established in Brussels, Belgium. Many TSHR-immunized mice developed TSAb (thyroid-stimulating antibody) and GO-like phenotypes. However, this disease model could not be reproduced in Cardiff, UK (Baker et al., 2005). Because both animal facilities in Brussels and Cardiff are not pathogen-free, environmental microbial factors are possible reasons that TSHR-induced GO could not be generated in Cardiff. This result was the first scientific evidence suggesting that gut microbiota may be related to GD/GO pathogenesis (Baker et al., 2005; Masetti and Ludgate, 2020). The commonly used GD/GO mouse models are generally BALB/c female mice, induced by electroporation of DNA plasmids expressing human TSHR A-subunit (Moshkelgosha et al., 2013) or injection of adenovirus expressing human TSHR A-subunit (Ad-TSHR289) (Chen et al., 2006). Both electroporation and Ad-TSHR289 can successfully induce GO-like phenotypes (Zhao et al., 2011).

#### GD/GO Mouse Models in Two Locations (Essen and London)

**Figure 1:**
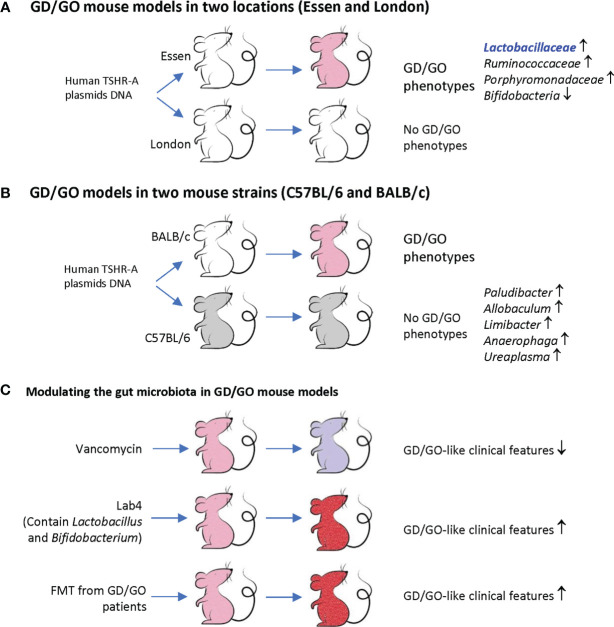
Animal model of Graves’ disease (GD)/Graves’ orbitopathy (GO) and gut microbiota. **(A)** BALB/c female mice were immunized with human TSHR-A plasmid DNA. The mice in Essen but not in London developed GD/GO phenotypes (pink). These mice have different gut microbiome profiles. Note, Essen mice have more *Lactobacillaceae*, which is also increased in GD patients and a component in Lab4. **(B)** BALB/c and C57BL/6 female mice were immunized with human TSHR-A plasmid DNA. Only BALB/c but not C57BL/6 female mice developed GD/GO phenotypes (pink). These mice have different gut microbiome profiles. **(C)** Modify the gut microbiota of TSHR immunized mice (pink) by antibiotic vancomycin, probiotic Lab4, and fecal material transfer (FMT) from GD/GO patients, resulting in changed GD/GO-like clinical features (light purple or red). Derived from Berchner-Pfannschmidt et al. (2016); Masetti et al. (2018); Moshkelgosha et al. (2018); Su et al. (2020), and Moshkelgosha et al. (2021).

A recent study investigated the TSHR plasmid-immunized mice in two locations (Essen in Germany and London in the UK) by 16S rRNA gene sequencing and routine microbiological tests (Berchner-Pfannschmidt et al., 2016; Masetti et al., 2018). These female BALB/c mice showed different gut microbiota compositions between these two SPF (specific pathogen-free) facilities. Essen mice had more abundant *Lactobacillaceae*, *Ruminococcaceae*, and *Porphyromonadaceae*, but did not have *Bifidobacteria*. The *Firmicutes : Bacteroidetes* (F/B) ratio changed in TSHR mice of both locations. Orbital adipogenesis in Essen mice was correlated positively with *Firmicutes* OTUs (operational taxonomic units) and negatively with *Bacteroidetes* phyla. Disease-associated taxonomies have been identified and explained the clinical differences observed between Essen and London (**Figure 1A**). These findings suggest that gut microbiota may modulate the clinical heterogeneity of GD/GO in TSHR-immunized mice (Masetti et al., 2018).

#### GD/GO Models in Two Mouse Strains (C57BL/6 and BALB/c)

After being immunized with TSHR plasmids, female C57BL/6 mice had produced both TSAb and TSBAb (TSH stimulating blocking antibody). However, none of these mice had GD or any orbital soft tissue changes, while BALB/c female animals showed GD and GO-like phenotypes. Splenic T cells isolated from C57BL/6 mice did not grow upon TSHR stimulation and mainly produced IL-10, but not proinflammatory cytokines such as IFN-γ (Moshkelgosha et al., 2018). 16S rRNA sequencing revealed increased beta-diversity between BALB/c and C57BL/6J gut microbiome and differential abundance of five genera (*Paludibacter*, *Allobaculum*, *Limibacter*, *Anaerophaga*, and *Ureaplasma*). These two mice strains had different correlations between gut microbiota and clinical manifestations; for instance, TSAb in C57BL/6J mice was correlated negatively with increased *Limibacter*. These results indicate that gut microbiota can modulate the immune activity, which explains different thyroid/orbit changes in different inbred mouse strains receiving TSHR immunization (**Figure 1B**).

#### Modulating the Gut Microbiota in GD/GO Mouse Models

To investigate whether the above-observed correlation indicates causation, the same research team altered the microbiota composition before the TSHR immunization by antibiotic vancomycin, probiotic Lab4, and fecal material transfer (FMT) from GO patients (Moshkelgosha et al., 2021). The antibiotic vancomycin was administered through drinking water, and probiotic Lab4 and FMT powder were administered through gavage. Vancomycin reduced the richness and diversity of gut microbiota. It depleted *Firmicutes* genera but increased *Bacteroides*, thus reducing the F/B ratio. It also significantly reduced CD4^+^CD25^+^ regulatory T cells (Tregs) in orbital lymph nodes and GD/GO-like clinical features. TSHR mice receiving FMT from GO patients had a similar microbiota composition with their donors at the early stage after the transfer. GD-like features and orbital brown adipose tissue (BAT) volumes increased after FMT.

Lab4 are lactic acid bacteria that have been isolated from the gut flora of healthy humans. It combines four strains of lactic acid bacteria, including two strains of *Lactobacillus acidophilus*, one *Bifidobacterium animalis* subsp. *lactis*, and a *Bifidobacterium bifidum*. Despite Lab4 containing two *Bifidobacteria* species, none of them can be detected in any mice fed with Lab4, and the reason was unknown. Lab4 increased Tregs in orbital lymph nodes only in mice without TSHR immunization but not in mice receiving TSHR immunization. Lab4 exacerbated TSHR-induced autoimmune GD and GO-like phenotypes. Thus, although Lab4 increases orbital Tregs in normal mice, it cannot prevent TSHR-induced tolerance breakdown (Moshkelgosha et al., 2021).

In another BALB/c mouse model injected with Ad-TSHR289, FMT from GD patients before TSHR immunization also increased total T4, thyrotropin receptor antibody (TRAb), and IL-17A and doubled the GD incidence (Su et al., 2020). These findings suggest that the gut microbiota is involved in the development of GD/GO-like phenotypes (**Figure 1C**).

In summary (**Table 1** and **Figure 1**), experimental GD/GO models of the same mouse strain but housed in two different animal facilities (Masetti et al., 2018), or different mouse strains from the same animal facility (Moshkelgosha et al., 2018), revealed significant differences in gut microbiota composition which explain variations in clinical manifestations. Modulating gut microbiota can change the incidence and severity of GD/GO (Su et al., 2020; Moshkelgosha et al., 2021). These results uncovered a crucial role of gut microbiota in initiating and developing GD/GO mouse models (Masetti and Ludgate, 2020).

### Changes of the Gut Microbiome in GD/GO Patients

There have been more than 10 clinical observational studies since 2018 comparing the gut microbiota of GD/GO patients to healthy controls (HCs). In total, fecal samples from 293 GD patients, 33 GO patients, and 271 healthy controls have been analyzed by 16S rRNA gene sequencing (**Table 1**).

#### Gut Microbiota in GD Patients

**Figure 2:**
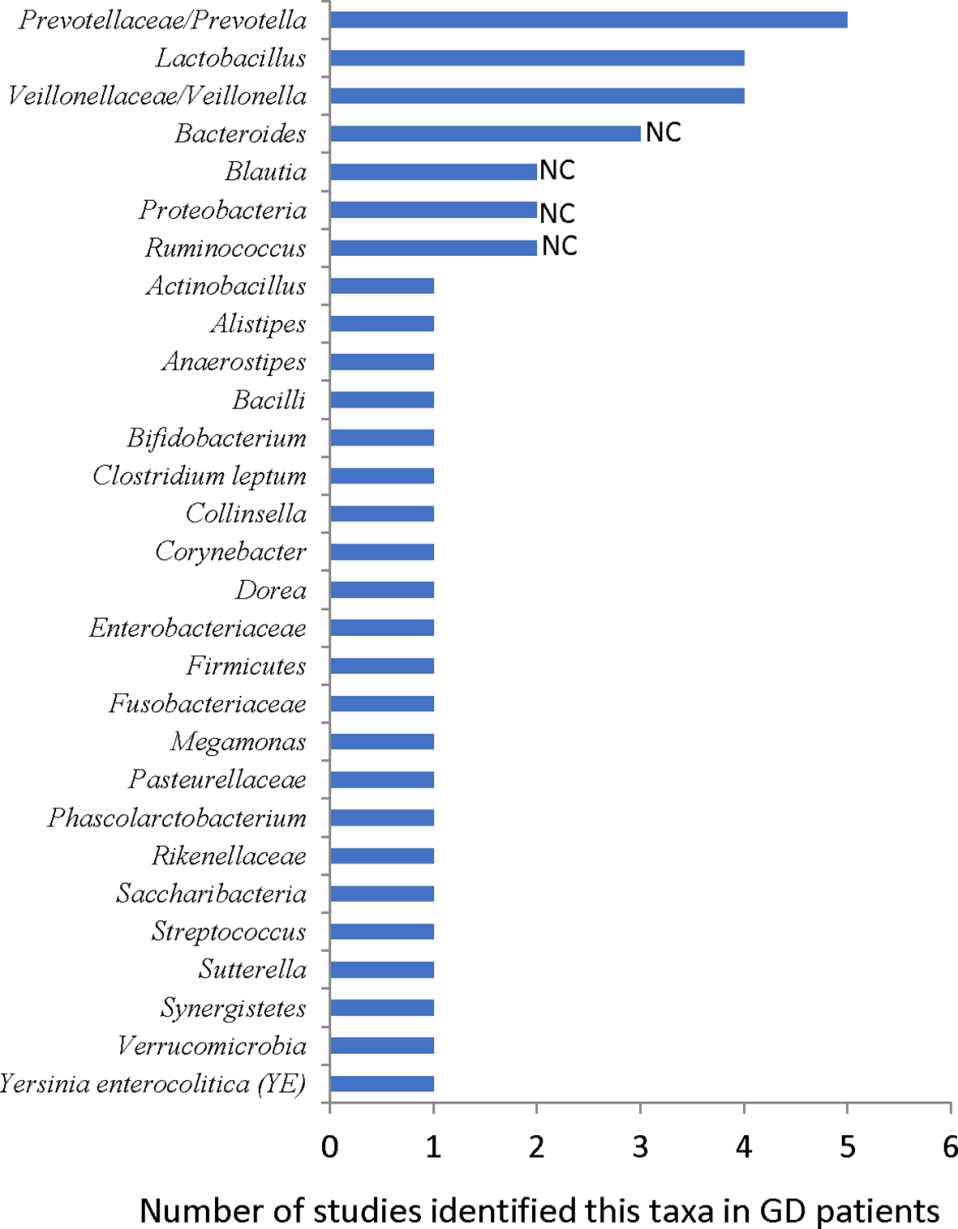
Reported gut microbiota taxa changed in GD patients. NC: results are not consistent between studies.

Until July 2021, there are eight papers, including 263 GD/239 HC samples, studying the gut microbiome of GD patients (**Table 1**). Overall, about 29 taxa are reported as differentially represented in GDs compared with HCs (**Figure 2**), and the gut microbial diversity decreased in most studies (Ishaq et al., 2018; Yang M. et al., 2019; Cornejo-Pareja et al., 2020; Su et al., 2020; Yan et al., 2020; Chang et al., 2021; Chen et al., 2021; Jiang et al., 2021). However, other results across these studies lack consistency. Differences in sample size, subject heterogeneity, study design, geographical location, and sequencing platform may contribute to the lack of reproducibility. Despite these limitations, several taxa were identified in three or more studies, including *Prevotellaceae* and *Veillonellaceae* at the family level and *Bacteroides*, *Lactobacillus*, *Prevotella*, and *Veillonella* at the genus level (**Figure 2**).

In a study comparing 27 GD patients and 11 HCs, *Prevotellaceae* increased in GD patients (Ishaq et al., 2018). In another study with 58 GD patients and 63 HCs, the random forest analysis showed that GD patients could be distinguished from HCs with 85% accuracy by three bacteria species, including *Prevotella* (Su et al., 2020). *Prevotella* also increased in GD patients in another study (Yan et al., 2020). Furthermore, *Prevotellaceae* and *Prevotella* were identified as the core microbiome of the GD group (Cornejo-Pareja et al., 2020). Moreover, they were found to be closely associated with GD patients (Chang et al., 2021). *Prevotella* has been linked with rheumatoid arthritis (RA), as specific antigens of *Prevotella* can shape or amplify immune responses in RA joints (Scher et al., 2013; Pianta et al., 2017; Pianta et al., 2021).

At the genus level, *Bacteroides* of GD patients decreased in two studies (Ishaq et al., 2018; Su et al., 2020) but increased in another one (Jiang et al., 2021). The *Firmicutes* and *Bacteroidetes* phyla are the major components of the human and mouse microbiota. The F/B ratio of their abundance is often used as a dysbiosis marker. The F/B ratio increased in GD patients in two studies (Ishaq et al., 2018; Yang M. et al., 2019) but decreased in another two (Chang et al., 2021; Jiang et al., 2021), suggesting that this index may not be related to the pathogenesis of GD.

In three studies, *Lactobacillus* increased in GD patients (Yan et al., 2020; Chen et al., 2021; Jiang et al., 2021). Interestingly, the TRAb level was correlated positively with *Lactobacillus*, and the abundance of *Lactobacillus* decreased after oral methimazole (MMI) treatment (Chen et al., 2021). The TSHR plasmid-immunized GD/GO mice in Essen also had more abundant *Lactobacillaceae* (**Figure 1**) (Berchner-Pfannschmidt et al., 2016; Masetti et al., 2018). *Lactobacillus* is generally not pathogenic in the human body, and it is a recognized element in some probiotics such as Lab4 (Moshkelgosha et al., 2021). However, some studies also suggested that *Lactobacillus* can induce macrophages to secrete proinflammation cytokines, such as IL-6 and TNF-α (Rocha-Ramírez et al., 2017), and the abundance of *Lactobacillus* increased in autoimmune hepatitis (Wei et al., 2020) and Crohn’s disease (Wang et al., 2014; Effenberger et al., 2021). These results are consistent with the observed effects of Lab4 on mice. Lab4 increases orbital Tregs in normal mice but exacerbates TSHR-induced autoimmune GD and GO-like phenotypes (**Figure 1**) (Moshkelgosha et al., 2021).

The abundance of the family *Veillonellaceae* or the genus *Veillonella* increased in GD patients (Yan et al., 2020; Chang et al., 2021; Chen et al., 2021). *Veillonella* is a symbiotic bacterium in humans and can become a conditional pathogenic bacterium. For instance, *Veillonella* acts as a pathogen in various inflammatory diseases such as pneumonia (Shah et al., 2008). *Veillonella* is also associated with the disease activity of autoimmune hepatitis (Wei et al., 2020) and recurrent Crohn’s disease (De Cruz et al., 2015). The role of *Prevotella*, *Lactobacillus*, and *Veillonella* in the pathogenesis of GD needs further investigation.

#### Gut Microbiota in GO Patients

There are fewer studies regarding the gut microbiota of GO patients. Until July 2021, there are only three reports from a single institute, including 30 GD, 33 GO, and 30 HC samples (**Table 1**). In one study, 33 active GO patients and 32 HCs were compared (Shi et al., 2019b). Community diversity decreased in GO patients, consistent with most observations of GD patients (Ishaq et al., 2018; Yang M. et al., 2019; Cornejo-Pareja et al., 2020; Su et al., 2020; Chen et al., 2021; Jiang et al., 2021). The F/B ratio decreased in the GO group, consistent with some recent findings in GD patients (Su et al., 2020; Chang et al., 2021; Jiang et al., 2021). Metabolic-network-driven analysis of 31 hyperthyroid GO patients indicated that TRAb was associated with *Prevotellaceae* OTUs, CAS (clinical activity score) was associated with *Bacteroides* OTUs, and thyroglobulin autoantibodies were associated with OTUs from the *Bacteroides stercoris* species (Shi et al., 2019a). They also compared the original GO cohort (33 GO/32 HCs) with 30 GD patients taking antithyroid drugs. They identified bacterial phyla with different abundances between GD and GO patients, including *Deinococcus–Thermus*, *Cyanobacteria*, *Chloroflexi*, and *Actinobacteria*. At the genus level, *Blautia*, *Anaerostipes*, *Dorea*, and *Butyricicoccus* were more abundant in the GD group, while *Subdoligranulum* and *Bilophila* were more abundant in the GO group. Random forest analysis could differentiate GO patients, GD patients, and HCs with 70%–80% accuracy. *Deinococcus–Thermus*, *Cyanobacteria*, and *Chloroflexi* were the major taxa determining the classification accuracy (Shi et al., 2021). These results need to be confirmed with more studies on larger groups of patients.

In summary, clinical studies concluded that GD/GO patients and healthy controls have a much different gut microbiota composition. Whether these gut microbiota alterations in GD/GO patients could contribute to disease pathogenesis or are just a consequence remains unknown. However, FMT from GD/GO patients significantly increased GD/GO incidence in the GD/GO mouse model, suggesting a fundamental pathogenic role of gut microbiota in the development of GD/GO (Su et al., 2020; Moshkelgosha et al., 2021).

## The Effects on the Gut Microbiota of Therapeutic Agents for GD/GO

The primary goal in the treatment of GD is restoring normal thyroid hormone levels. To reach this goal, antithyroid drugs (such as thionamides), radioiodine, and thyroidectomy are commonly used. The treatment of GO is stage-dependent. The anti-inflammatory agent is recommended for active progressive disease, and rehabilitative surgery is performed only in the stable inactive stage (Davies et al., 2020). Glucocorticoids (GCs) are the first-line treatment; if an insufficient response is observed after 6 weeks, second-line therapy, including immunosuppressants (such as mycophenolate mofetil and azathioprine) and biologic agents (such as rituximab, infliximab tocilizumab, and teprotumumab) should be considered (Davies et al., 2020; Taylor et al., 2020). Recent studies indicated that some of these therapeutic agents (including antithyroid drugs, glucocorticoids, immunosuppressants, and biologics) could also affect the gut microbiota (**Table 2**).

### Antithyroid Drugs and Gut Microbiota

Methimazole (MMI) and propylthiouracil (PTU) are the commonly used antithyroid drugs (ATDs). ATD can change the gut microbiota structure in wild-type adult rats (Shin et al., 2020; Sun et al., 2020) and GD patients (Sun et al., 2020; Chen et al., 2021; Huo et al., 2021).

The effects of PTU on microbiota were studied in adult male SD rats (Shin et al., 2020). After treatment for 4 weeks, the alpha-diversity did not change. However, *Christensenellaceae*, *Tenericutes*, and *Mollicutes* increased, while *Ruminococcaceae*, *Prevotella*, *Mogibacteriaceae*, *Alcaligenaceae*, *Betaproteobacteria*, *Burkholderiales*, *Sutterella*, and *Ruminococcus* decreased (Shin et al., 2020). The effects of MMI/PTU on microbiota were also measured in female adult SD rats (Sun et al., 2020). ATDs increased the alpha-diversity, different from the results in female rats (Shin et al., 2020). The ATD group had more *Bacteroidetes*, *Proteobacteria*, and *Spirochaetae*, but fewer *Firmicutes* at the phylum level and more *Prevotellaceae* and *Ruminococcaceae* but fewer *Lactobacillaceae* and *Peptostreptococcaceae* at the family level. Compared with the MMI group, the PTU group had more *Spirochaetae* at the phylum level and more *Spirochaetaceae* and *Clostridiaceae_1* but less *Lachnospiraceae* and *Rikenellaceae* at the family level. The microbial dysbiosis index (MDI) increased after ATD treatment, indicating that the gut microbiota structure was disturbed in the treatment group. The MDI of the MMI group was higher than that of the PTU group (Sun et al., 2020).

ATDs can also affect the gut microbiota composition of GD patients. The fecal samples from 20 MMI-treated GD patients, 20 PTU-treated GD patients, and 50 healthy controls were analyzed by 16S rRNA sequencing. The MMI group had a higher Ace index than the PTU group. The community diversity was different between the two drug treatment groups. The MMI group had more *Firmicutes* at the phylum level, while the PTU group had more *Bacteroidetes.* The MMI group had more *Blautia* and *Escherichia–Shigella* at the genus level, while the PTU group had more *Bacteroides* and *Lachnoclostridium.* The MDI and the F/B ratio suggested that dysbiosis occurred in both drug-treated groups. Interestingly, ATD treatment reduced short-chain fatty acid (SCFA)-producing bacteria, including *Faecalibacterium*, *Ruminococcaceae*, *Lactobacillus*, and *Blautia* (Sun et al., 2020).

In a recent study with 15 GD patients, MMI treatment reduced the abundance of *Blautia*, *Lactobacillus*, and *Streptococcus* but increased *Proteobacteria* (Chen et al., 2021). In another study with 8 GD patients, MMI treatment for 6 months also reduced the microbial Shannon index and *Faecalibacterium prausnitzii*, *Ligilactobacillus salivarius*, *Lactococcus lactis*, and some species of the genera *Porphyromonas* and *Prevotella* (Huo et al., 2021).

### Glucocorticoids and Gut Microbiota

GCs are the first-line treatments for moderate to severe active GO. GCs are potent immune-modulating drugs with a number of side effects, such as GC-induced obesity or osteoporosis, but little is known about the effect of steroid treatment on gut microbiota in GO patients. In patients with GC-induced obesity, gut microbial diversity decreased, *Firmicutes* (e.g., genus *Streptococcus*) increased, and *Bacteroidetes* were depleted. Concomitantly, the SCFA level decreased in gut microbial metabolites of these patients (Qiu et al., 2019).

In animal models, GCs can change the composition of gut microbiota. In mice, dexamethasone increased the abundance of *Actinobacteria*, *Bifidobacterium*, and *Lactobacillus* compared with controls (Huang et al., 2015). A recent study found different results regarding *Lactobacillus* (Zhao et al., 2020). At the phyla level, short-term dexamethasone treatment increased the abundance of *Firmicutes.* At the genus level, dexamethasone increased the abundance of *Lachnospiraceae*, *Oscillibacter*, *Ruminococcaceae*, *Ruminiclostridium*, *Anaerotruncus*, and *Butyricicoccus* but reduced the abundance of *Lactobacillus*, *Enterorhabdus*, and *Pseudomonas* (Zhao et al., 2020).

It is also proven in mice that subcutaneous prednisolone implants for 8 weeks can promote dysbiosis, cause intestinal barrier leaks, and raise serum endotoxin levels. GCs reduced *Verrucomicobiales* and *Bacteriodales* and increased *Clostridiales.* These effects mediated the GC-induced osteoporosis (Schepper et al., 2020). In birds (yellow-legged gull *Larus michahellis*), corticosterone implants reduced *Mycoplasma* and *Microvirga*, which were potentially pathogenic avian bacteria, and increased *Firmicutes*, which was beneficial for birds (Noguera et al., 2018).

GCs can also alter the gut microbiota through changes in brain function. Stress can increase serum corticosteroid levels; stress also changes the mouse microbiome, and it reduces intestinal *Bacteroides* while increasing the relative abundance of bacteria in the genus *Clostridium* (Bailey et al., 2011). This effect is similar to the results of long-term subcutaneous prednisolone implants (Schepper et al., 2020).

### Immunosuppressant Drugs Have a Direct Effect on Microbiota

Recent clinical trials concluded that combining steroids with immunosuppressant drugs (azathioprine and mycophenolate mofetil) has beneficial effects for GO patients (Kahaly et al., 2018; Rajendram et al., 2018). While these effects are generally attributed to their immunosuppressive activity, azathioprine (AZA) and mycophenolate mofetil (MMF) directly affect the microbiota. In liver cells, AZA is metabolized to 6-mercaptopurine (MP), which can inhibit the maturation of B and T lymphocytes and the synthesis of DNA/RNA and proteins in immune cells. AZA can inhibit the growth of *Campylobacter concisus*, *Bacteroides fragilis*, *Bacteroides vulgatus*, *Escherichia coli*, and *Mycobacterium Avium paratuberculosis* (Shin and Collins, 2008; Antoniani et al., 2013; Liu et al., 2017). AZA is commonly used to treat inflammatory bowel disease (IBD). AZA can restore intestinal microbial diversity in patients with Crohn’s disease by decreasing *Proteobacteria* but increasing *Bacteroidetes* (Effenberger et al., 2021).

MMF is an inhibitor of inosine-5′-monophosphate dehydrogenase (IMPDH). MMF inhibits both T-cell and B-cell activities by blocking purine synthesis and is widely used in organ transplant recipients to reduce immune rejection (Ritter and Pirofski, 2009). MMF has some antibacterial (such as *Staphylococcus epidermidis*), antifungal (such as *Cryptococcus*, *Aspergillus*, *Pneumocystis jirovekii*, and *Candida albicans*), and antiviral (such as *Camelpox virus*, *Cowpox virus*, *Monkeypox virus*, and *Vaccinia virus*) activities, as it inhibits the synthesis of microbial DNA/RNA (Jones, 2020).

MMF treatment can cause gastrointestinal (GI) toxicity in organ transplant recipients. In mice, MMF reduced the overall gut microbial diversity and increased *Proteobacteria*. MMF-induced GI toxicity could be reversed or prevented using broad-spectrum antibiotics and was absent in germ-free animals (Flannigan et al., 2018; Taylor et al., 2019). Spontaneously hypertensive rats (SHR) are commonly used hypertension animal models. They have decreased gut microbial richness and acetate- and butyrate-producing bacteria and increased F/B ratio and lactate-producing bacteria (Yang T. et al., 2019). MMF can reduce blood pressure in SHR (Rodríguez-Iturbe et al., 2002). MMF triggered substantial changes in the SHR microbiota taxa and reduced gut dysbiosis by reducing the F/B ratio and lactate-producing bacteria and increasing acetate- and butyrate-producing bacteria (Robles-Vera et al., 2021).

### Biological Agents and Gut Microbiota

Several biological agents that can be used as a novel therapy for GO patients. Even though there were very few studies regarding the effects of these agents on gut microbiota, their underlying pathways can interact with gut microbiota. For example, rituximab (RTX), a chimeric human-murine anti-CD20 monoclonal antibody, has been used to treat active moderate–severe GO for more than 15 years (Vannucchi et al., 2021). RTX eliminates orbital and peripheral B cells, therefore, reducing the production of antibodies. B-cell-produced IgA is the most abundant antibody in the mucosa; secretory IgA (SIgA) is secreted into the lumen of the gut. IgA can bind to multiple distinct taxonomic groups of the microbiota and is involved in the elimination, neutralization, and colonization of gut microbiota. IgA can also regulate bacterial gene expression (Weis and Round, 2021). Thus, RTX should have some effects on gut microbiota, but this has not been specifically addressed.

Tocilizumab, a humanized recombinant IL-6R monoclonal antibody, can improve disease activity and severity in corticosteroid-resistant GO patients (Perez-Moreiras et al., 2018; Sánchez-Bilbao et al., 2020). Infliximab, an antitumor necrosis factor (TNF)-α antibody, has been successfully used to treat sight-threatening GO (Durrani et al., 2005; Komorowski et al., 2007; Fallahi et al., 2021). Both IL-6 and TNF-α are monocyte-derived key proinflammation cytokines. Gut microbiota can modulate the production of these cytokines through their metabolites. The microbial tryptophan metabolite tryptophol potently influences cytokine production, which has strong inhibitory effects on the TNF-α response. On the other hand, palmitoleic acid can affect the production of monocyte-derived cytokines (TNF-α, IL-1β, IL-6) but not lymphocyte-derived cytokines (IFN-γ, IL-17, IL-22) (Schirmer et al., 2016). *Lachnospiraceae* and *Ruminococcaceae* families, typically producing SCFAs, define the frequently relapsing disease and poor treatment response to anti-TNF-α in Crohn’s disease (Yilmaz et al., 2019). Like AZA, the anti-TNF-α antibody can restore intestinal microbial diversity in patients with Crohn’s disease by decreasing *Proteobacteria* but increasing *Bacteroidetes* (Effenberger et al., 2021). In 20 enteropathic arthritis patients who followed a Mediterranean diet, anti-TNF-α antibody treatment for 6 months could increase *Lachnospiraceae* family and *Coprococcus* genus and also induce a decreasing trend in *Proteobacteria* and *Gammaproteobacteria* and an increasing trend in *Clostridia* (Ditto et al., 2021).

Teprotumumab, an insulin-like growth factor-1 receptor (IGFR-1) inhibiting monoclonal antibody, was approved in the USA to treat GO (Kahaly et al., 2021). Gut microbiota can modulate circulating IGF-1 in the host. Microbiota-derived metabolites such as SCFAs are sufficient to induce IGF-1 (Yan and Charles, 2018). How teprotumumab affects gut microbiota has not yet been explored.

In summary, the fact that ATDs, GCs, AZA, and MMF can change the microbiota composition is consistent with the notion that modifying the gut microbiota may reduce the severity of GD/GO, thus strengthening the concept that GD/GO and dysbiosis are tightly connected. The relationship between biological agents and gut microbiota needs further study in the future.

## How Gut Microbiota Affect GD/GO Development

**Figure 3:**
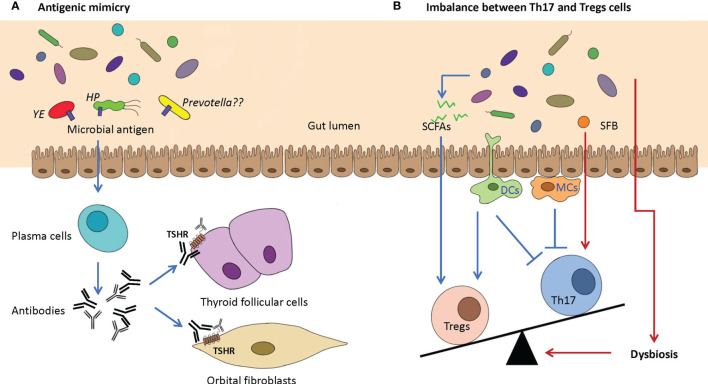
Two potential proposed mechanisms of GD/GO are caused by dysbiosis of the gut microbiome. **(A)** Antigenic mimicry. Antigenic mimics in the gut microbiome, which have a highly similar structure or sequence with the autoantigens (e.g., TSHR), could activate plasma cells to produce antibodies that can bind TSHR on the thyroid follicular cells and orbital fibroblasts. Possible pathogenic microbes include *Yersinia enterocolitica* (YE), *Helicobacter pylori* (HP), and *Prevotella*. **(B)** Imbalance between Th17 and Tregs cells. Intestinal dysbiosis may cause the absence of beneficial microbiota and the reduction in beneficial anti-inflammatory metabolites such as SCFAs, which can increase the production of Tregs. SFB can promote the differentiation and maturation of Th17 cells. The imbalance between Th17 and Tregs cells can indirectly promote the progression of GD/GO. TSHR, thyroid-stimulating hormone receptor; SCFA, short-chain fatty acids; SFB, segmented filamentous bacteria.

Two major mechanisms are proposed, namely, molecular/antigenic mimicry and imbalance of proinflammation T helper 17 cells (Th17) and Treg cells (**Figure 3**).

### Antigenic Mimicry

*Yersinia enterocolitica* (YE) and *Helicobacter pylori* (HP) were thought to be possible environmental risk factors for GD for a long time (Wolf et al., 1991). The structural or conformational similarity between different antigens can lead to cross-reactivity, also known as molecular or antigenic mimicry. Cross-reactivity between gut microbial peptides and self-antigens can produce autoreactive T cells and induce autoimmunity (Avni and Koren, 2018; Rojas et al., 2018; Wildner and Diedrichs-Möhring, 2020). If microbes have similar protein structures in their host, they will escape immune detection (**Figure 3A**).

Such similarities can be shared by amino acid and nucleotide sequence or protein 3D structures (Miraglia and Colla, 2019). YE porin proteins have sequence similarity with TSHR and can stimulate B cells to produce autoantibodies to TSHR (Wang et al., 2010; Hargreaves et al., 2013). The IgG from YE-infected patients can induce GD-like changes in human thyroid structures, and GD patients have a higher YE infection rate than the healthy controls (Wolf et al., 1991). Although YE is considered involved in the development of GD, 16S rRNA gene sequencing did not detect it in GD patients. However, PCR analysis could easily detect YE in GD patients with diarrhea (Su et al., 2020).

HP in the human gastric mucosa can also affect the development of GD/GO (Bassi et al., 2010). Human TSHR is partly aligned with nine HP proteins such as NADH dehydrogenase subunit L, ABC transporter, and radical SAM protein (Figura et al., 2019). The most virulent HP strains express the cytotoxin-associated gene A antigens (CagA). GD patients have a much higher prevalence of CagA^+^ HP than healthy controls (Bassi et al., 2010). While these clinical observations support that antigenic mimicry may trigger GD/GO, it has not been proven in any animal model yet.

Several recent studies revealed that *Prevotellaceae* and *Prevotella* consistently increase in GD patients (Ishaq et al., 2018; Cornejo-Pareja et al., 2020; Su et al., 2020; Yan et al., 2020; Chang et al., 2021). *Prevotella* has been linked with rheumatoid arthritis (RA), as specific antigens of *Prevotella*, such as Pc-27 and *N*-acetylglucosamine-6-sulfatase (GNS), can trigger antigenic mimicry with RA joints (Scher et al., 2013; Pianta et al., 2017; Pianta et al., 2021). If these *Prevotella-*related antigens have similarities with human TSHR, they need further investigation.

### Imbalance Between Th17 and Treg Cells

The gut-associated lymphoid tissue has many T-cell populations, including proinflammation helper T (Th) cells and anti-inflammation Tregs. T helper cells include Th1, Th2, and Th17 cells. Th17 cells are the most important autoimmunity-related cells. Autoimmune diseases are closely related to abnormal Th17 cells (Bettelli et al., 2007; Pandiyan et al., 2019). Usually, Th17 and Tregs cells are in a dynamic balance to maintain the immune homeostasis of the gut mucosa. Gut microbiota can keep the balance between Th17/Tregs (Omenetti and Pizarro, 2015) (**Figure 3B**).

For instance, some gut microorganisms, such as *Intestinimonas* and *Roseburia*, can produce SCFAs, including primarily acetic acid, propionic acid, and butyric acid. SCFAs can increase Tregs in the gut mucosa (Smith et al., 2013). *Segmented filamentous bacteria* (SFB) promote the differentiation and maturation of Th17 cells (Ivanov et al., 2008; Hedblom et al., 2018). Gut microbiota can affect gut dendritic cells and macrophage cells. Gut dendritic cells can secrete transforming growth factor-beta (TGF-β), promoting the differentiation of Th17 and Treg cells. Dendritic cells also produce retinoic acid, which can promote Treg development but suppress Th17 cells. Gut macrophages can inhibit Th17 development by producing IL-2 and IL-10 (Masetti and Ludgate, 2020). Thus, dysbiosis may increase Th17 cells and suppress Treg production, which can cause many intestine inflammatory conditions and some extraintestinal diseases such as autoimmune uveitis (Zhuang et al., 2017).

As expected, GD patients have much less circulating CD4^+^Foxp3^+^ Tregs but more CD4^+^IL-17^+^ Th17 cells (Qin et al., 2017; Su et al., 2020). Gas chromatography–mass spectrometry (GC–MS) analysis generated metabolic profiles of gut microbiota in GD patients and indicated that two important SCFAs (propionic acid and butyric acid) were significantly decreased in GD patients (Su et al., 2020). SCFA-producing *Bacteroides fragilis* YCH46 strain (B.f.S) was significantly reduced in GD patients. The culturing medium of B.f.S increased Tregs and IL-10 levels but reduced Th17 cells and IL-17A levels in peripheral blood mononuclear cells (PBMCs) from healthy individuals. B.f.S also exacerbated the imbalance of Treg/Th17 cells in GD patients (Su et al., 2020). GO patients also have much more Th17 cells and higher IL-17A expression than normal controls (Fang et al., 2016). These results are further confirmed by single-cell sequencing of retrobulbar tissues from GO patients (Fang et al., 2019). Single-cell sequencing identified six major cell clusters in retrobulbar tissues, including orbital fibroblasts and lymphocytes. In the CD4^+^CD8^−^ T-cell subset, both IFN-γ–producing and IL-17A–producing T cells increased, but FoxP3^+^ Tregs decreased in GO orbits (Fang et al., 2019). Thus, increased Th17 and impaired Treg responses may be involved in GD/GO pathogenesis.

Removing CD4^+^CD25^+^ Tregs afforded some GD-resistant C57BL/6 mice susceptible to Ad-TSHR289 immunization and increased the GD severity in susceptible BALB/c mice. Removing CD4^+^CD25^+^ Tregs also promoted TSAB production but suppressed thyroid-blocking antibody synthesis. These results indicate that Tregs are essential for Ad-TSHR289–induced GD phenotypes (Saitoh and Nagayama, 2006). However, the importance of Th17 cells in the immune response of the GD mouse model varies among different genetic backgrounds (Horie et al., 2011). BALB/c and NOD-H2(h4) mouse strains with wild-type (WT) or knockout (KO) IL-17 genes were immunized with Ad-TSHR289. BALB/c mice developed GD with WT or KO IL17 gene, but NOD-H2(h4) mice developed GD only in some animals with WT IL17 gene. This result suggested that IL-17 is essential for the GD development only in NOD-H2(h4) but not in BALB/c background (Horie et al., 2011).

These findings suggest that Th17/Treg imbalance is involved in developing GD/GO in some genetic backgrounds or ethnic groups (for instance, all these abovementioned clinical observations are from the Asian population).

## Targeting the Gut Microbiota to Treat GD/GO

Dysbiosis is closely related to the development of GD/GO; therapeutic approaches targeting the gut microbiota may provide potential benefits to GD/GO patients. Currently, antibiotics, probiotics, diet modifications, and fecal microbial transplantation are the four major strategies proposed.

### Antibiotics

Antibiotics can change the gut microbiome. It was shown that oral antibiotic vancomycin could reduce the GD/GO severity in mouse models by reducing gut microbiota richness and diversity (**Figure 1C**). The reduced orbital pathology was correlated positively with *Akkermansia* (Moshkelgosha et al., 2021). As HP infection of the gastric mucosa is associated with GD through an increased inflammatory status and molecular mimicry, anti-HP therapy may also benefit GD/GO patients (Figura et al., 2019).

### Probiotics

Probiotics are live microorganisms with health benefits, which improve or restore the gut microbiota. Probiotics can promote the differentiation of Tregs, thus modifying the intestine immune homeostasis (Lin, 2019). Probiotics have been tested in GD/GO mouse models (Moshkelgosha et al., 2021) and GD patients (Huo et al., 2021).

The probiotic Lab4 is a consortium comprising of *Lactobacillus* and *Bifidobacterium*. Lab4 elevated the orbital CD25^+^ Treg cells but promoted the GD/GO phenotypes of TSHR-immunized mice (Moshkelgosha et al., 2021). One possible reason is that *Lactobacillus* in Lab4 may be pathogenic to GD as it frequently increases in fecal samples from GD patients (Yan et al., 2020; Chen et al., 2021; Jiang et al., 2021). These results also suggest that probiotics alone are not enough to suppress GD/GO development. Treatment with MMI and probiotic *Bifidobacterium longum* (2 × 10^7^ CFU per day) for 6 months improved thyroid function and significantly reduced the TRAb concentration of nine GD patients (Huo et al., 2021). This treatment increased *Bifidobacterium adolescentis*, *Bifidobacterium angulatum*, *Bifidobacterium breve*, *B. longum*, and *Faecalibacterium prausnitzii* and reduced *Blautia hansenii*, *Clostridium estertheticum*, and *Klebsiella pneumoniae.* Several microbial metabolic pathways were enriched in subjects receiving the probiotic *B. longum* treatment, including fatty acid biosynthesis, toluene degradation, phenylacetate degradation, and flavin biosynthesis. SCFAs also increased in these patients (Huo et al., 2021). The above results from animal models and GD patients support the idea that *Lactobacillus* may not benefit GD patients.

Future studies need to optimize the beneficial microbe stains in the probiotics formula; for instance, the formula should not include *Lactobacillus*, *Prevotella*, and *Veillonella*, as their abundance often increases in GD patients (Ishaq et al., 2018; Yang M. et al., 2019; Cornejo-Pareja et al., 2020; Su et al., 2020; Yan et al., 2020; Chang et al., 2021; Chen et al., 2021; Jiang et al., 2021).

### Diet Modifications and Selenium Supplements

The diet can shape the microbiome composition. Culture and geographic-related diet differences can cause microbiome composition changes, for instance, *Firmicutes* enriched in the USA and Russia, *Bacteroides* spp. enriched in France and China, and *Prevotella* spp. enriched in Germany and India (Fröhlich and Wahl, 2019). Most GD patients have a reduced microbial diversity, which can be helped by a fiber-rich, low-calorie diet (Meijnikman et al., 2018). Diet with more vegetables increases SCFAs and *Bifidobacteria*, but animal fat increases the production of secondary bile acids (Ercolini and Fogliano, 2018). Diet with eicosapentaenoic acid (EPA) can inhibit IFN-γ and IL-17 productions, thus attenuating experimental autoimmune encephalomyelitis (EAE) (Unoda et al., 2013).

The Mediterranean diet (MD) is consumed in countries bordering the Mediterranean sea and is characterized by a high intake of vegetables and fruits, legumes, and whole grains combined with a moderate amount of red wine and olive oil. It is well known that MD plays a protective role in preventing cardiovascular diseases, type 2 diabetes mellitus, obesity, Alzheimer’s or Parkinson’s disease, and cancer. MD was associated with a higher abundance of *Bacteroidetes*, *Prevotellacea*, and *Prevotella* and a lower concentration of *Firmicutes* and *Lachnospiraceae*. MD can also induce fecal propionate and butyrate (De Filippis et al., 2016; Gutiérrez-Díaz et al., 2016). As discussed above, *Prevotellacea* and *Prevotella* often increase in GD patients (Yan et al., 2020; Chang et al., 2021; Chen et al., 2021), and MD may not be a good choice for GD/GO patients. However, these results are mainly from research in Asia; if MD has any beneficial effects on GD/GO patients in the other geographic locations needs further study.

In the 2021 European Group on Graves’ orbitopathy (EUGOGO) clinical practice guidelines for the medical management of GO, oral selenium supplementation is recommended for patients with mild GO (Bartalena et al., 2021). Mild GO patients from selenium-deficient areas can benefit from oral selenium supplementation. A double-blind, randomized clinical trial confirmed that sodium selenite could improve both the quality of life and overall ocular outcome and slow the progression in mild GO patients (Marcocci et al., 2011). These effects may be partially related to the role of selenium on gut microbiota. In adult C57BL/6 male mice, selenium in the diet can increase the microbiota diversity (Kasaikina et al., 2011), increase *Turicibacter* and *Akkermansia*, but reduce *Mucispirillum* (Qixiao Zhai et al., 2018). *Turicibacter* has been reported to display potential anti-inflammatory activities in the gut, and *Akkermansia* plays an essential role in the gut barrier protection, immune modulation, and metabolic regulation of the host (Qixiao Zhai et al., 2018). *Akkermansia* is also correlated positively with reduced orbital pathology in vancomycin-treated GO mouse models (**Figure 1C**) (Moshkelgosha et al., 2021). Thus, selenium supplementation increased the gut microbial diversity and positively modulated health beneficial microbes and negatively modulated the deleterious microbes in mice models. However, the effects of selenium supplementation on human gut microbiota are still unknown (Ferreira et al., 2021).

### Fecal Microbiota Transplantation

Fecal microbiota transplantation (FMT) transfers fecal bacteria and other microbes from a healthy donor into the patient to replace their dysbiotic microbiota. FMT has been successfully used to treat *Clostridium difficile* infection in colitis by increasing the diversity of the host microbiota (Cheng et al., 2019). FMT has also been used to treat rheumatoid arthritis (Zeng et al., 2021). FMT from GD/GO patients can increase the severity of induced GD/GO-like features in TSHR immunization mouse models (Su et al., 2020; Moshkelgosha et al., 2021), but if FMT from healthy donors can suppress established GD/GO manifestation has not been tested yet.

## Conclusions and Future Directions

The relationship between gut microbiota and GD/GO has been uncovered during the past 4 years. Oral antibiotic vancomycin reduces disease severity in GD/GO mouse models, but FMT from GD/GO patients exaggerates the disease. There are significant differences in microbiota composition between GD/GO patients and healthy controls. *Lactobacillus*, *Prevotella*, and *Veillonella* often increase in GD patients. GCs are the first-line treatment for GO and can also change the composition of the microbiota. Two immunosuppression drugs (AZA and MMF) for GO have some antimicrobial properties; two AIDs (MMI and PTU) can change microbiota composition. Antigenic mimicry and imbalance of Th17/Tregs are likely the mechanisms for the effects of dysbiosis on GD/GO phenotypes.

Interventions including antibiotics, probiotics, and diet modification that modulate the gut microbiota have been actively investigated in preclinical models and clinical settings. However, only limited data exist on their effects on GD/GO patients. More research is needed to reveal molecular pathways linking gut and thyroid functions and how they impact orbital autoimmunity. For instance, the gut microbial features of GO patients need to be determined in more geographic locations; the effects of different probiotic formulas on GD/GO mouse model and patients need to be investigated; and how *Lactobacillus*, *Prevotella*, and *Veillonella* affect GD/GO phenotypes is still unknown. We believe microbiota-targeting therapeutics will be an important strategy in the management of GD/GO. This conclusion requires not only a thorough understanding of the distinct gut microbial composition and function of GD/GO patients but also carefully designed clinical trials.

## Author Contributions

JH, YT, YC, and DC conceived and designed the manuscript, and all authors wrote, edited, and approved the manuscript.

## Funding

This study was supported by grants to DC from the National Natural Science Foundation of China (81870665, 82171063).

## Conflict of Interest

The authors declare that the research was conducted in the absence of any commercial or financial relationships that could be construed as a potential conflict of interest.

## Publisher’s Note

All claims expressed in this article are solely those of the authors and do not necessarily represent those of their affiliated organizations, or those of the publisher, the editors and the reviewers. Any product that may be evaluated in this article, or claim that may be made by its manufacturer, is not guaranteed or endorsed by the publisher.
